# Cardiovascular risk factor changes associated with six-year circuit training in older adults: a retrospective cohort analysis

**DOI:** 10.3389/fragi.2026.1728121

**Published:** 2026-02-16

**Authors:** Marcelo Pereira de Lima, Renata Miyabara, Lujain Fouad Khalaf, Shady Salah Bagady, Saed Fawaz Raddawi, José Antônio Silva Júnior, Ovidiu Constantin Baltatu, Luciana Aparecida Campos

**Affiliations:** 1 Center of Innovation, Technology, and Education (CITE) at Anhembi Morumbi University – Anima Institute, Sao Jose dos Campos Technology Park, Sao Josedos Campos, Brazil; 2 UNINOVAFAPI University Center – Afya Educacional, Teresina, Piauí, Brazil; 3 College of Medicine, Alfaisal University, Riyadh, Saudi Arabia; 4 Postgraduate Program in Medicine, Universidade Nove de Julho (UNINOVE), Sao Paulo, Brazil

**Keywords:** aging population, health promotion, multidisciplinary approach, older people, physical exercise interventions

## Abstract

**Background:**

Physical exercise can significantly impact chronic disease prevention and health promotion in older adults. This retrospective cohort study evaluated the association between participation in a multidisciplinary physical exercise program and cardiovascular health outcomes in older adults over 6 years.

**Methods:**

The Active Life circuit resistance training program incorporated aerobic, strength, balance, and flexibility exercises delivered in a community-based setting. While the Active Life Program was prospectively planned and implemented as a 6-year community-based intervention, the present study is a retrospective analysis of participants selected based on adherence (≥75% attendance) and availability of complete data from a digital platform. Thirty participants (n = 30; mean age 70.2 ± 5.4 years) completed the program, with 30 sedentary controls (n = 30) included for comparison. Eligibility criteria included age ≥60, presence of chronic cardiovascular risk factors, and ability to engage in moderate physical exercise. A concurrent sedentary control group (attendance <25%) was selected from the same eligible population. Cardiovascular risk factors were assessed at 6-month intervals.

**Results:**

Compared to the sedentary group, the Active Life group demonstrated significantly lower systolic arterial pressure (p = 0.0009, η^2^ = 11.53%), with an average between-group difference of 10.5 ± 2.4 mmHg over the 1.5–6.0-year period. A significant reduction in the triglyceride-to-HDL-C ratio was observed starting at 2.5 years (p = 0.0146, η^2^ = 7.09%). Additionally, the Active Life group exhibited lower triglyceride levels (p = 0.0134, η^2^ = 5.73%; average difference: 33.9 ± 5.8 mg/dL) and fasting glucose levels (p = 0.0163, η^2^ = 6.23%; average difference: 16.1 ± 4.9 mg/dL) over the 3.0–6.0-year period compared to controls. No significant differences were observed in diastolic blood pressure, total cholesterol, LDL-C, or HDL-C.

**Conclusion:**

This retrospective analysis suggests that sustained participation in a circuit resistance training program may be associated with favorable cardiovascular and metabolic profiles in older adults. However, given the non-randomized design, small sample size, and potential selection bias, these findings should be considered preliminary and exploratory. Future randomized controlled trials are needed to confirm these associations and establish causality.

## Introduction

As people age, the risk of chronic diseases such as cardiovascular disease, diabetes, and obesity increases. Physical inactivity is a major risk factor for these conditions, and older adults are particularly susceptible to the negative effects of a sedentary lifestyle. However, the potential impact of physical activity on chronic disease prevention and health promotion in older adults is well established. Compelling evidence supports that regular physical activity can improve cardiovascular fitness, muscle strength, and bone density, as well as reduce the risk of chronic diseases, cognitive decline, and functional limitations. Physical exercise is presently the only proven means to reduce the accelerated decline in muscle strength exhibited by older adults. However, its effect is hindered by low adherence rates, even under well-structured programs.

Circuit resistance training is one effective means to enhance muscle strength in older and middle-aged adults since it is less strenuous and time-consuming exercise and it may be implemented in healthy and metabolically challenged older adults (Buch et al., 2017). This type of exercise program typically includes resistance exercises that target multiple muscle groups, with minimal rest periods between exercises. The intensity of the exercises is controlled by means of an effort perception scale, setting the intensity range from moderate to strong as optimum.

Several studies have shown that both resistance training and aerobic endurance exercise can have a positive impact on cardiovascular risk factors including systolic arterial pressure (Roberson et al., 2018), and dyslipidemia (Leitão et al., 2021). However, results have been inconsistent due to training characteristics, frequencies, and lengths of the physical exercise programs (Hazley et al., 2010; Mendes et al., 2014; Weston et al., 2014).

Circuit resistance training programs for older adults usually last for 6–12 months, with an average frequency of two–three sessions per week. Circuit resistance training may increase adherence to training in older adults because of its shorter duration and lower intensity than traditional resistance training programs (Buch et al., 2017).

Despite the clear benefits of PA for older adults, many face barriers that make it difficult for them to be physically active, such as mobility issues, lack of access to safe exercise environments, and lack of support. This highlights the need for strategies to increase PA levels in older adults and improve their overall health and wellbeing.

This study aimed to evaluate the association between participation in a circuit resistance training program (CRTP) and cardiovascular health outcomes in older adults over a 6-year period. We hypothesized that CRTP, which offers a shorter and lower-intensity alternative to traditional resistance training, may be associated with improved adherence and favorable long-term cardiovascular outcomes in older adults. Given the observational nature of this retrospective cohort analysis, we sought to generate preliminary evidence to inform future randomized controlled trials.

## Methods

### Study design

The Active Life Program was prospectively implemented as a 6-year community-based intervention. For this study, we conducted a retrospective cohort analysis of participants who demonstrated adherence to the program and for whom complete data were available in the Loggi digital platform.

### Ethics statement

This study was approved by the Anhembi Morumbi University Ethical Committee (CAAE: 11818919.8.0000.5492). The study was carried out following the resolutions 466/2012 and 340/2004 of the National Health Council (Ministry of Health) for research on human subjects and with international medical ethics guidelines (Geneva Declaration, International Code of Medical Ethics, 1948, amended 1983).

The studies involving humans were approved by The study protocol (CAAE: 11818919.8.0000.5492) was approved by the Ethics Committee at Anhembi Morumbi University. The studies were conducted in accordance with the local legislation and institutional requirements. The participants provided their written informed consent to participate in this study. Written informed consent was obtained from the individual(s) for the publication of any potentially identifiable images or data included in this article.

### Active life program description

The Active Life Program, aimed at promoting physical exercise in older adults, was a multidisciplinary effort involving physical education professionals, nurses, physicians, and nutritionists. The team provided motivational support, monitored progress, and tailored exercise programs to participants' needs and abilities. Physicians provided medical oversight, health assessments, and personalized advice. Nutritionists provided nutritional advice and support. IT specialists maintained technological solutions to monitor progress and facilitate communication between the Active Life team and participants.

### Physical exercise intervention program

The physical exercise program aligns with elements of circuit resistance training following the American College of Sports Medicine standards, incorporating aerobic, muscle strengthening, and flexibility activities (ChodzkoZajko et al., 2009). The Active Life program, which consisted of a circuit resistance training program, targeted cardiovascular, metabolic, and neuromuscular adaptation in older people (RomeroArenas et al., 2013). The circuit was performed in an alternating system, with each exercise performed continuously for 1 minute, followed by 1 minute of recovery. This format of circuit resistance training may have contributed to the program’s effectiveness (MarcosPardo et al., 2019), especially in the cardiovascular system (RomeroArenas et al., 2013).

A gradual and planned exercise training program consisted of 50–60 min per session at a rate of perceived effort (RPE) of one–3, repeated 2-3 times per week. The training sessions included a variety of aerobic, strength, balance, and flexibility exercises led by a physical therapist and a physical education instructor. The training session began with a general warm-up (articular and cardiorespiratory), 10-min mats, and flexibility exercises. The second phase included a 20-min cardiovascular exercise in the form of a group walk around the gymnasium’s sports court, with intensities ranging from mild to moderate and respecting each participant’s limits. The following phase was to strengthen the lower and upper extremities with 20 min of alternating workouts with canes, dumbbells, ankle weights, and elastic bands. The exercise concluded with a 10-min stretch of the major muscle groups, with two replications of 30 s per muscle group. Competitions were held once a week to help group members develop their socializing and teamwork skills. These competitions were a circuit of static and dynamic workouts involving identical motions required to execute fundamental everyday chores, game perception, and body awareness. The ability of participants to socialize and enjoy group exercise, as defined by McPhate et al., was a significant positive component of our physical exercise program (McPhate et al., 2016).

### Outcome measures

The primary outcome measures were cardiovascular risk factors. The timeline for outcome assessments was at 6-month intervals of the exercise intervention program. Outcome assessors were masked to the group allocation. The following cardiovascular risk measures were investigated: arterial blood pressure, triglycerides, HDL, LDL, total cholesterol, fasting glycemia, and body mass index (BMI). Blood pressure was measured in the non-dominant arm, with the individual seated and resting for at least 5 min. Body weight was obtained on an electronic scale with the individual wearing only light clothing and with an empty bladder. Height was obtained using a wall stadiometer with the individual barefooted, and the body mass index (BMI = weight/height^2^) was calculated. Biochemical examinations were performed in a certified clinical laboratory. Blood samples were collected in the morning (between 7:00 and 9:00 a.m.) after an overnight fast of at least 8 h. Participants were instructed to avoid vigorous physical activity and alcohol consumption for 24 h prior to data collection. Participants traveled to the clinical laboratory by their own means; no specific transportation instructions were provided. Baseline demographic and health data were collected from Unimed’s patient database. Follow-up measurements, including arterial pressure, lipid profiles, and fasting glucose levels, were scheduled to monitor the participants' progress throughout the study.

### Study participants’ recruitment and retention

Recruitment was conducted through direct, face-to-face interactions with the Active Life team, who acted as facilitators to establish trust and credibility. To promote sustained engagement, the program incorporated social activities both during and outside exercise sessions. A flexible attendance policy allowed for absences of up to 1 month per year, accommodating holidays and personal commitments, thereby fostering a participant-centered and sustainable environment.

Participant data were systematically managed using Loggi, a digital platform developed to import and organize healthcare and registration data (ForMedici Soluções, 2025). This platform enabled efficient tracking of participant engagement and facilitated communication between the multidisciplinary health team and participants, supporting adherence throughout the study.

Retention rates were monitored retrospectively from each participant’s date of enrollment, with follow-up data obtained via anonymized chart review. Only participants with complete baseline and follow-up data were included in the final analysis. Adherence to the Active Life Program was assessed over the entire selected time period, not just the first year, to ensure that only those with meaningful and sustained exposure to the intervention were analyzed.

### Participants in the study–eligibility, inclusion, and exclusion criteria

This study is a retrospective cohort analysis of a prospectively implemented, community-based physical exercise intervention in older adults. Participants were not randomized; instead, group allocation was determined retrospectively based on program adherence and data completeness over the selected 6-year period.

Eligibility criteria included: age 60 years or older; presence of chronic cardiovascular risk factors (specifically, essential hypertension, type II diabetes, or obesity); an up-to-date patient profile in the Unimed system with available arterial pressure, lipid profiles, and fasting glucose data; and the ability to engage in moderate physical activity. Eligible participants were also required to be insufficiently active, defined as not meeting the World Health Organization’s recommendations for physical activity (i.e., less than 30 min per day of moderate activity on 5 days per week, or less than 30 min per day of vigorous activity on 3 days per week).

Inclusion criteria were voluntary participation and willingness and ability to engage in moderate forms of physical activity. Exclusion criteria comprised any disease limiting physical activity (such as primary neuromuscular disease, cancer, or recent major surgery), psychological disease, or prescription of neuroleptic medication. Group allocation was determined retrospectively: The intervention group included only those who attended at least 75% of scheduled exercise sessions and had complete baseline and follow-up data. The sedentary (control) group consisted of eligible individuals who attended less than 25% of scheduled sessions during the selected time period and had complete data records. This approach ensured that the control group represented a truly sedentary comparison (adherence <25%) and allowed for contemporaneous comparison between groups.

### Statistical analysis

The association between participation in the physical exercise intervention program and cardiovascular risk factors was evaluated using a repeated measures (RM) two-way analysis of variance (ANOVA) based on the general linear model. Before analysis, all variables met normality assumptions for RM two-way ANOVA as verified by Shapiro-Wilk and Kolmogorov-Smirnov tests. Group (Active Life vs. Sedentary) served as the between-subjects factor and time (assessment intervals at 6-month intervals from baseline to 6 years) served as the within-subjects factor. For the RM two-way ANOVA, matched values were organized as both stacked and spread across a row, allowing for proper handling of the repeated-measures structure within GraphPad Prism.

Šídák’s multiple comparisons test was applied using a single pooled variance estimate. This approach adjusts for multiple pairwise comparisons across timepoints within each outcome variable while maintaining appropriate statistical power. Separate ANOVAs were conducted for each outcome measure (systolic blood pressure, diastolic blood pressure, triglycerides, HDL, LDL, total cholesterol, fasting glucose, triglyceride/HDL ratio, and BMI). To facilitate interpretation beyond statistical significance, effect sizes are reported as eta-squared (η^2^), representing the proportion of total variance explained by each factor. This measure is appropriate for ANOVA designs and indicates the practical significance of the observed effects. Effect sizes were interpreted according to conventional guidelines: small (η^2^ ≈ 1%), medium (η^2^ ≈ 6%), and large (η^2^ ≈ 14%) (Cohen, 1973; Richardson, 2011). Eta-squared values are reported as percentages in the results for ease of interpretation. Given the final sample size of 30 participants per group, the study was adequately powered (>80% at α = 0.05) to detect large effects (η^2^ ≥ 14%) but may have been underpowered to detect small-to-moderate effects.

Only participants with complete data across all 13 timepoints (baseline plus 12 follow-up assessments at 6-month intervals) were included in the analysis (complete case approach). No imputation was performed for missing data. This approach may introduce bias favoring participants with higher adherence and engagement, which is acknowledged as a limitation.

The analysis was conducted using GraphPad Prism version 10.1.1 for Mac OS X (GraphPad Software, La Jolla California USA, www.graphpad.com). The data are represented as the mean ± standard error of the mean (SEM). A two-tailed p-value <0.05 was considered statistically significant.

## Results

**FIGURE 1:**
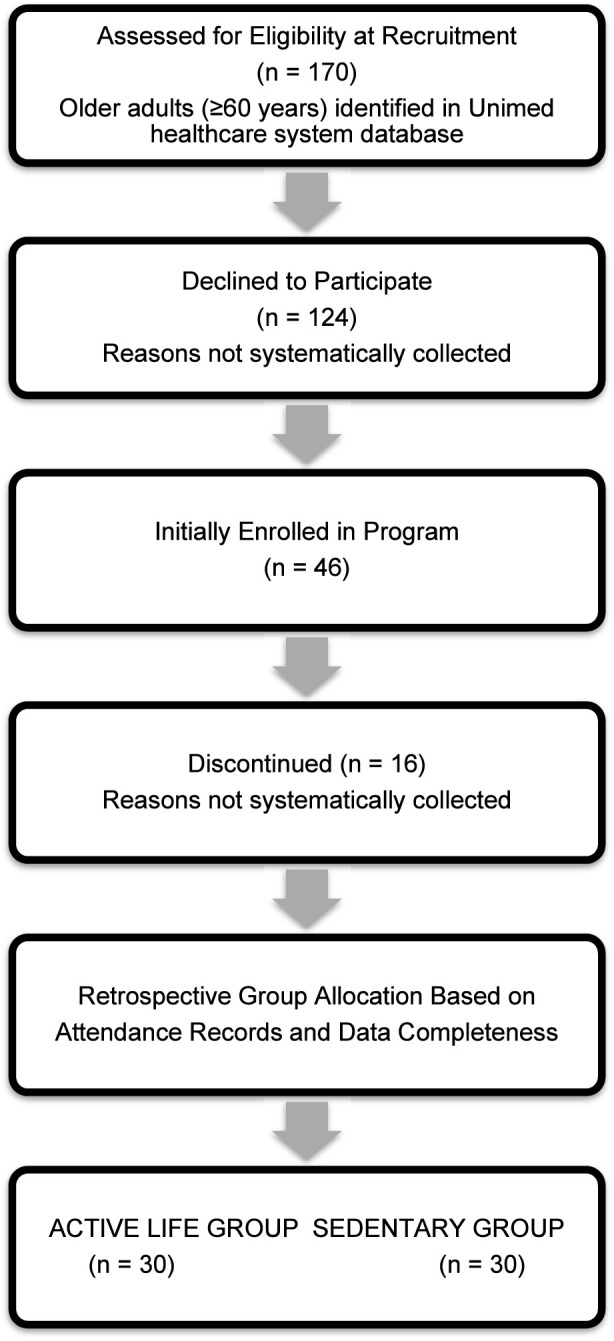
Participant flow diagram for the retrospective cohort analysis.

Of the 170 patients identified as eligible during the recruitment period, 124 (73%) declined participation (Figure 1). Of the 46 who initially enrolled, 16 (35%) discontinued during the 6-year study period. Reasons for dropout were not systematically collected. As identified in the Loggi digital platform database, 6 years after the program’s inception, a group of thirty participants (15 women and 15 men) remained committed to the program, completed the study, and systematically delivered the outcome measures. A concurrent group of eligible patients (13 women and 17 men) who did not consent to engage in the Active Life program but had their medical records thoroughly monitored served as a control, sedentary group.

The cohort of study participants (Active Life and sedentary groups) had an average age of 70.2 ± 5.4 years. No significant differences were found between the sexes regarding average age or the impacts of the Active Life program. Consequently, the effects of the Active Life program were collectively presented for each group, including both sexes. The baseline levels of the investigated outcome measures demonstrated similar levels, except for BMI, which exhibited a significant difference between the Active Life and sedentary groups (Table 1). Additionally, no effects of the Active Life program were observed on BMI.

**TABLE 1:** Baseline characteristics of study participants. Data are mean ± SEM. η^2^ calculated from one-way ANOVA for continuous variables; — indicates not applicable for categorical variables. *p < 0.05.

Characteristic	Active life (n = 30)	Sedentary (n = 30)	p-value	η^2^ (%)
Age, years	69.5 ± 1.0	70.9 ± 1.0	0.34	1.6
Female, n (%)	15 (50%)	13 (43%)	0.59	—
BMI, kg/m^2^	28.5 ± 0.2	32.6 ± 0.4	0.0008*	17.8
Systolic BP, mmHg	123.0 ± 2.2	126.7 ± 1.9	0.21	2.7
Diastolic BP, mmHg	77.0 ± 1.6	79.3 ± 1.4	0.28	2.0
Triglycerides, mg/dL	134.1 ± 10.0	143.3 ± 14.3	0.60	0.5
HDL, mg/dL	46.7 ± 2.0	44.6 ± 2.1	0.48	0.9
Triglycerides/HDL	3.0 ± 0.3	3.8 ± 0.6	0.27	2.1
LDL, mg/dL	121.5 ± 6.3	123.4 ± 7.3	0.84	0.1
Total cholesterol, mg/dL	192.9 ± 6.4	196.1 ± 7.9	0.76	0.2
Fasting glucose, mg/dL	106.1 ± 3.7	108.4 ± 3.9	0.66	0.3

**FIGURE 2:**
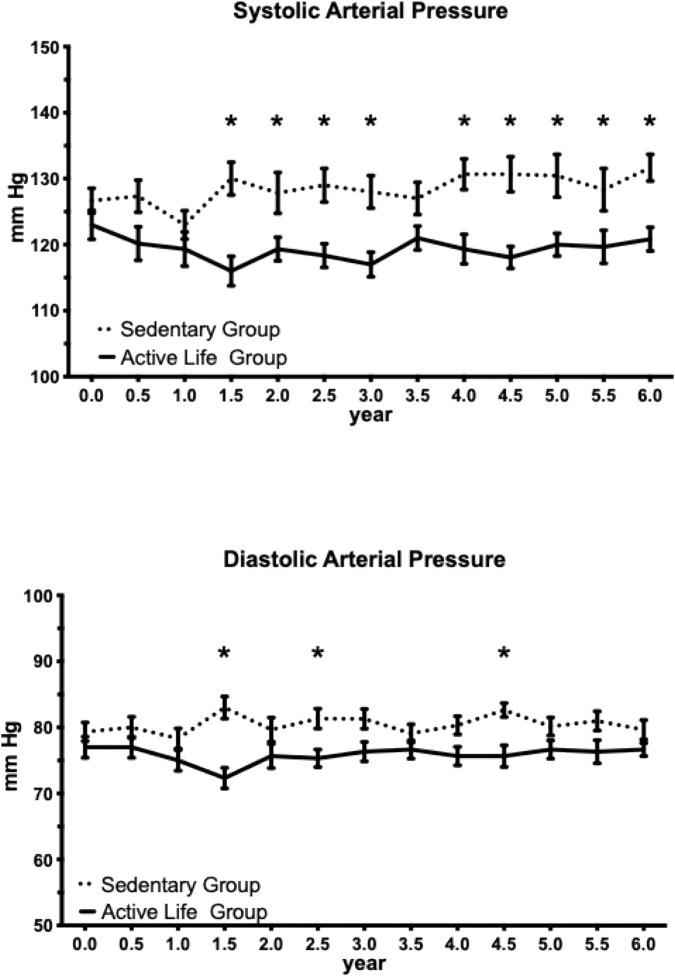
Association between the Active Life program and blood pressure over 6 years. Impact of the physical exercise program on systolic blood pressure (SBP) and diastolic blood pressure (DBP) in the Active Life group compared to the sedentary control group. Data are presented as mean ± SEM. SBP was significantly lower in the Active Life group compared to controls from 1.5 years onward, with an average between-group difference of 10.5 ± 2.4 mmHg over the 1.5–6.0-year period. No significant sustained differences were observed for DBP. *p < 0.05 for between-group comparison at the indicated timepoint (repeated-measures two-way ANOVA with Šídák’s multiple comparisons test using single pooled variance). SBP, systolic blood pressure; DBP, diastolic blood pressure; mmHg, millimeters of mercury; SEM, standard error of the mean.

The Active Life group exhibited significantly lower systolic blood pressure compared to the Sedentary group (main effect of group: F (1, 29) = 13.68, p = 0.0009, η^2^ = 11.53%), with the group factor accounting for 11.53% of total variance. The main effect of time was not significant (F (12, 348) = 1.12, p = 0.34, η^2^ = 0.92%), nor was the group × time interaction (F (12, 348) = 1.06, p = 0.39, η^2^ = 0.96%), indicating that the between-group difference remained relatively stable over time rather than progressively widening. Šídák-corrected post-hoc comparisons identified significant between-group differences emerging at 1.5 years and sustained through 6.0 years, with an average difference of 10.5 ± 2.4 mmHg (mean ± SD) favoring the Active Life group over this period (Figure 2).

The Active Life group also demonstrated significantly lower diastolic blood pressure compared to the Sedentary group (main effect of group: F (1, 29) = 11.27, p = 0.0022, η^2^ = 7.26%), with the group factor accounting for 7.26% of total variance. Similar to systolic pressure, neither the main effect of time (F (12, 348) = 0.50, p = 0.91, η^2^ = 0.50%) nor the group × time interaction (F (12, 348) = 1.47, p = 0.13, η^2^ = 1.67%) reached statistical significance. However, in contrast to systolic blood pressure, Šídák-corrected post-hoc comparisons revealed significant between-group differences at fewer individual timepoints. This pattern suggests that while the Active Life program was associated with lower diastolic blood pressure overall, the magnitude of the difference at any single timepoint was smaller and more variable than observed for systolic blood pressure (Figure 2).

**FIGURE 3:**
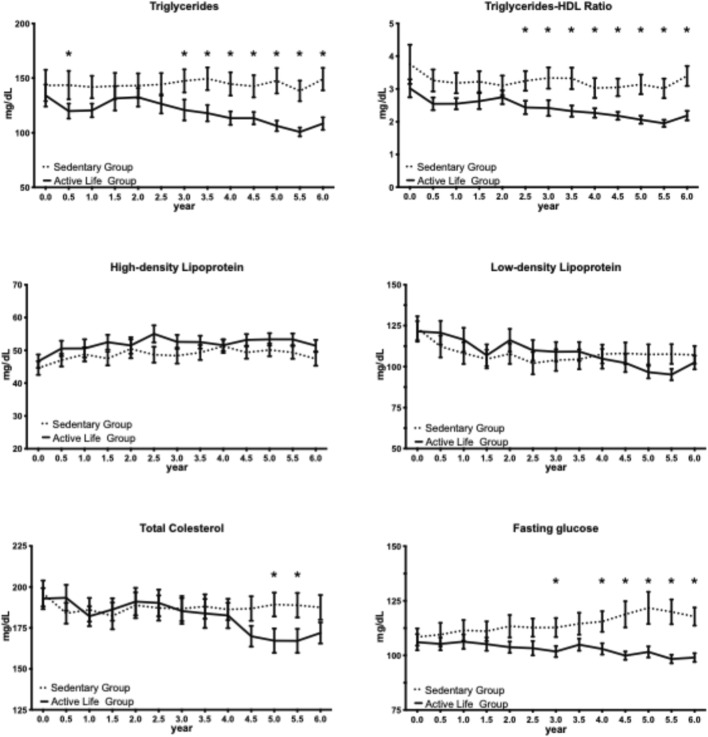
Association between the Active Life program and metabolic parameters over 6 years. Impact of the physical exercise program on triglycerides, triglyceride-to-HDL ratio, high-density lipoprotein (HDL), low-density lipoprotein (LDL), total cholesterol, and fasting glucose in the Active Life group compared to the sedentary control group. Data are presented as mean ± SEM. Significant between-group differences favoring the Active Life group were observed for: triglycerides (from 3.0 years; average difference 33.9 ± 5.8 mg/dL), triglyceride-to-HDL ratio (from 2.5 years; average difference 0.96 ± 0.15), and fasting glucose (from 3.0 years; average difference 16.1 ± 4.9 mg/dL). No significant sustained differences were observed for HDL, LDL, or total cholesterol. *p < 0.05 for between-group comparison at the indicated timepoint (repeated-measures two-way ANOVA with Šídák’s multiple comparisons test using single pooled variance); mg/dL, milligrams per deciliter; SEM, standard error of the mean.

The triglycerides-HDL-C ratio was significantly reduced by the Active Life program starting at 2.5 years from its commencement, with an average difference of 0.96 ± 0.15 (mean ± SD) over the 2.5–6.0-year period compared to the sedentary group (Figure 3). Additionally, the program resulted in a substantial average difference of 33.9 ± 5.8 (mean ± SD) in triglyceride levels over the 3.0–6.0-year period and an average difference of 16.1 ± 4.9 (mean ± SD) in fasting glucose levels over the same timeframe, both in comparison to the sedentary group (Figure 3). The other outcomes, including diastolic arterial pressure, total cholesterol, LDL, and HDL, exhibited comparable levels between the Active Life and sedentary groups, with only isolated significant differences observed.

Lipid profile and metabolic outcomes showed significant differences between the Active Life and Sedentary groups across several parameters over a 6-year study period (Figure 3; Table 2). Triglyceride levels in the Active Life group were significantly lower than in the Sedentary group (main effect: F (1, 29) = 6.94, p = 0.0134, η^2^ = 5.73%), with a significant interaction indicating worsening triglycerides in the Sedentary group as time progressed (group × time interaction: F (12, 348) = 1.93, p = 0.0296, η^2^ = 1.00%). This trend was marked by an average difference of 33.9 ± 5.8 mg/dL observed from 3.0 to 6.0 years. The triglyceride-to-HDL ratio also favored the Active Life group (main effect: F (1, 29) = 6.75, p = 0.0146, η^2^ = 7.09%) while showing stable between-group differences from 2.5 years onward. For HDL levels, a significant change was observed over time (F (12, 348) = 4.60, p < 0.0001, η^2^ = 1.83%), but no group differences (main effect: F (1, 29) = 1.64, p = 0.21, η^2^ = 1.88%) or interaction were noted. Total cholesterol did not differ significantly between groups (F (1, 29) = 0.47, p = 0.50, η^2^ = 0.49%), yet both time effects and group × time interactions were significant, indicating varying trajectories throughout the study. LDL followed a similar pattern, with no overall group effect (F (1, 29) = 0.004, p = 0.95, η^2^ < 0.01%) but significant changes over time (F (12, 348) = 6.54, p < 0.0001, η^2^ = 2.91%) suggesting differential trajectories between groups. In terms of metabolic outcomes, fasting glucose levels were lower in the Active Life group (main effect: F (1, 29) = 6.50, p = 0.0163, η^2^ = 6.23%), with a significant interaction indicating widening differences over time (group × time: F (12, 348) = 3.22, p = 0.0002, η^2^ = 1.83%). Overall, the Active Life group’s glucose levels remained stable or decreased, contrasting with increasing levels in the Sedentary group, with significant disparities emerging by 3.0 years (average difference: 16.1 ± 4.9 mg/dL).

**TABLE 2:** Repeated-measures two-way ANOVA results for all cardiovascular and metabolic outcomes.

Outcome	Source	F (DFn, DFd)	p-value	η^2^ (% Variance)	Effect size
Systolic BP	Time	F (12, 348) = 1.12	0.342	0.92	Small
	Group	F (1, 29) = 13.68	0.0009*	11.53	Large
	Interaction	F (12, 348) = 1.06	0.390	0.96	Small
Diastolic BP	Time	F (12, 348) = 0.50	0.913	0.50	Small
	Group	F (1, 29) = 11.27	0.0022*	7.26	Medium-large
	Interaction	F (12, 348) = 1.47	0.134	1.67	Small
Triglycerides	Time	F (12, 348) = 1.56	0.103	0.92	Small
	Group	F (1, 29) = 6.94	0.0134*	5.73	Medium
	Interaction	F (12, 348) = 1.93	0.0296*	1.00	Small
HDL	Time	F (12, 348) = 4.60	<0.0001**	1.83	Small
	Group	F (1, 29) = 1.64	0.211	1.88	Small
	Interaction	F (12, 348) = 0.91	0.538	0.43	Small
TG/HDL ratio	Time	F (12, 348) = 3.45	<0.0001**	1.89	Small
	Group	F (1, 29) = 6.75	0.0146*	7.09	Medium-large
	Interaction	F (12, 348) = 1.05	0.404	0.52	Small
Total cholesterol	Time	F (12, 348) = 3.15	0.0003**	1.44	Small
	Group	F (1, 29) = 0.47	0.499	0.49	Negligible
	Interaction	F (12, 348) = 2.91	0.0007**	1.45	Small
LDL	Time	F (12, 348) = 6.54	<0.0001**	2.91	Small
	Group	F (1, 29) = 0.004	0.948	<0.01	Negligible
	Interaction	F (12, 348) = 2.29	0.0081*	1.12	Small
Fasting glucose	Time	F (12, 348) = 0.52	0.904	0.26	Negligible
	Group	F (1, 29) = 6.50	0.0163*	6.23	Medium
	Interaction	F (12, 348) = 3.22	0.0002**	1.83	Small

The Active Life group (n = 30) was compared with the sedentary control group (n = 30) using repeated-measures two-way ANOVA, with group as the between-subjects factor and time (13 assessments at 6-month intervals) as the within-subjects factor. Three effects are tested for each outcome: Time (change over assessment periods), Group (overall difference between Active Life and sedentary participants), and Interaction (whether groups changed differently over time). A significant interaction indicates diverging or converging trajectories between groups. Abbreviations: BP, blood pressure; DF, degrees of freedom; DFn, numerator degrees of freedom; DFd, denominator degrees of freedom; *η* ^*2*^, eta-squared (proportion of variance explained); HDL, high-density lipoprotein cholesterol; LDL, low-density lipoprotein cholesterol; TG, triglycerides. ***p < 0.05, **p < 0.01, ***p < 0.001, ***p < 0.0001.

The Active Life circuit resistance training program was associated with significant between-group differences in multiple cardiovascular and metabolic risk factors (Table 2). Significant main effects of group were observed for systolic blood pressure (p = 0.0009, η^2^ = 11.53%), diastolic blood pressure (p = 0.0022, η^2^ = 7.26%), triglycerides (p = 0.0134, η^2^ = 5.73%), triglyceride-to-HDL ratio (p = 0.0146, η^2^ = 7.09%), and fasting glucose (p = 0.0163, η^2^ = 6.23%), all favoring the Active Life group. Significant group × time interactions were observed for triglycerides (p = 0.0296, η^2^ = 1.00%) and fasting glucose (p = 0.0002, η^2^ = 1.83%), indicating that the between-group differences for these outcomes widened progressively over time—a pattern consistent with the Active Life program preventing the metabolic deterioration observed in sedentary controls. For total cholesterol and LDL, significant interactions were observed (p = 0.0007, η^2^ = 1.45% and p = 0.0081, η^2^ = 1.12%, respectively) despite non-significant main effects of group. This pattern indicates differential trajectories between groups that balanced out over the full study period rather than consistent between-group differences. HDL was the only lipid parameter showing no significant group effect or interaction, with both groups following similar trajectories over time (significant time effect only, p < 0.0001, η^2^ = 1.83%).

## Discussion

The findings of this retrospective cohort study suggest that sustained participation in a 6-year circuit resistance training program may be associated with favorable cardiovascular and metabolic profiles in older adults. The Active Life group demonstrated lower systolic and diastolic blood pressure, triglycerides, triglyceride-to-HDL-C ratio, and fasting glucose compared to sedentary controls. The effect sizes for these outcomes ranged from medium to large, suggesting clinically meaningful differences beyond statistical significance. To our knowledge, this represents one of the longest follow-up periods for a community-based exercise intervention study in older adults reported in the literature.

Both systolic and diastolic blood pressure were lower in the Active Life group compared to sedentary controls, with significant overall group effects for both measures. However, the pattern of significance differed between measures. For systolic blood pressure, post-hoc analyses revealed sustained significant differences from 1.5 years through 6.0 years. In contrast, diastolic blood pressure showed significant differences at only isolated timepoints despite the significant overall effect, suggesting a smaller and more variable benefit. This pattern may reflect the smaller effect magnitude for diastolic pressure combined with greater measurement variability, or genuine physiological differences in how these measures respond to circuit resistance training. Meta-analytic evidence supports exercise-induced reductions in both systolic and diastolic pressure, though our findings suggest that sustained, detectable benefits may be more readily achieved for systolic pressure in community-based programs of this nature (Edwards et al., 2023; McDonagh et al., 2025; AzamiAghdash et al., 2025).

The Generation 100 trial, a 5-year randomized controlled trial evaluating exercise training effects in older adults (Letnes et al., 2022), provides an important comparator for long-term intervention studies. While our observational design precludes direct comparison, both studies suggest that sustained exercise participation over multiple years may be associated with favorable cardiovascular profiles in older adults. The non-significant interactions for blood pressure measures indicate that the between-group differences remained relatively stable over time. This pattern suggests that cardiovascular benefits may have been established relatively early and subsequently maintained, rather than continuing to accumulate throughout the intervention period.

The lipid parameters revealed distinct patterns of association with program participation. Triglycerides and the triglyceride-to-HDL-C ratio were consistently lower in the Active Life group. The progressive widening of between-group differences in triglyceride levels over time suggests that the intervention may have prevented the metabolic deterioration observed in sedentary controls. The triglyceride-to-HDL-C ratio is an established indicator of metabolic health and has been associated with incident type 2 diabetes and cardiovascular disease risk in older adults (Liu et al., 2022). In our previous short-term study, we observed that a 3-month physical exercise program reduced this ratio (Pereira de Lima et al., 2020). The present study extends these observations, demonstrating that such improvements may be sustained over 6 years with continued program participation. High-density lipoprotein levels did not differ between groups, suggesting similar trajectories without program-specific benefit, which contrasts with reports of modest exercise-induced HDL-C increases in prior meta-analytic work and may reflect the characteristics of our population or the intensity of the intervention (PalazónBru et al., 2021; Smart et al., 2025; Kelley and Kelley, 2006). Total cholesterol and low-density lipoprotein presented a more complex pattern, with differing trajectories between groups but no overall group differences, suggesting that any between-group differences at specific timepoints were offset by opposite differences at other timepoints and may be influenced by unmeasured confounders such as changes in lipid-lowering medication over the 6-year period that were not systematically documented (Albarrati et al., 2018; Dudum et al., 2019).

Fasting glucose was lower in the Active Life group, with the between-group difference widening progressively over time—the strongest temporal divergence observed among all outcomes, suggesting that the program may have mitigated the age- and inactivity-related deterioration in glucose regulation seen in older adults (Ryan, 2000; Jiang et al., 2022). These findings align with evidence that structured physical exercise is an effective non-pharmacological strategy for improving glycemic control in adults with type 2 diabetes, with training consistently associated with reductions in fasting glucose and HbA1c (Ribeiro et al., 2023; Hou et al., 2023). The progressive divergence in glucose levels between groups underscores the potential importance of sustained exercise engagement for maintaining long-term metabolic health in older adults (Celli et al., 2022; Kanaley et al., 2022).

A notable finding was the gradual emergence of significant between-group differences over time. For systolic blood pressure, differences emerged at 1.5 years; for the triglyceride-to-HDL ratio at 2.5 years; and for triglycerides and fasting glucose at 3.0 years. This temporal pattern aligns with clinical guidelines emphasizing the importance of sustained, long-term exercise engagement (ChodzkoZajko et al., 2009), even when initial short-term improvements may be modest. This gradual emergence of benefits has important clinical implications (Yu et al., 2025). Healthcare providers recommending exercise programs to older adults should counsel patients that meaningful improvements may take several years to manifest and that sustained engagement is essential for realizing potential benefits (Kunutsor and Laukkanen, 2024; Izquierdo et al., 2021).

The Active Life program faced significant challenges in participant recruitment, with a majority of eligible individuals declining participation. This finding is consistent with literature documenting barriers to physical activity engagement among older adults, including poor health literacy, socio-cultural factors, which affect physical activity in older people (Babak et al., 2022; Jayasinghe et al., 2016; Buchmann et al., 2023). Health literacy is essential for better health, longevity, and quality of life. Active learning programs have proven effective in promoting a healthy lifestyle among older adults with low health literacy (Stormacq et al., 2020; Uemura et al., 2021). To address these challenges and enhance participant engagement, the program incorporated social activities both during and outside the exercise sessions. The weekly competitions provided an opportunity for physical exercise in a group setting. This social component of the program aligns with evidence suggesting that group-based exercise may enhance adherence in older adults, though the specific social and psychological effects were not formally evaluated in this study (Arkkukangas et al., 2025). The Loggi platform enabled efficient tracking of participant engagement and facilitated communication between the health team and participants.

The multidisciplinary approach, involving physicians, physiotherapists, physical activity coaches, and IT specialists, has been found to effectively enhance adherence to exercise programs in older adults (ColladoMateo et al., 2021). The multidisciplinary team approach of the Active Life program facilitated the accommodation of individual needs and preferences. This approach aligns with evidence-based strategies for enhancing exercise adherence (André et al., 2024; ColladoMateo et al., 2021), though the specific contributions of individual program components to participant retention were not formally assessed in this study.

## Limitations

Several important limitations should be considered when interpreting these findings. The retrospective, non-randomized design precludes causal inference. The relatively high rate of participant refusal introduces the potential for selection bias. Participants who completed the 6-year program likely represent a highly motivated and potentially healthier subset of the eligible population (Jungreitmayr et al., 2022; Huberty et al., 2013). The absence of systematic data on reasons for non-participation and dropout limits our understanding of these biases. Previous literature suggests that completers may differ from non-completers in baseline fitness, health status, social support, and motivation (Overgaard et al., 2024; Shaw et al., 2022). Baseline BMI differed significantly between groups and was not adjusted for in the primary analysis. Higher baseline obesity in the control group may have contributed to their less favorable outcomes independent of exercise participation. The study did not systematically track participants’ diets or medications. Dietary changes from nutritionist involvement in the Active Life program and medication adjustments over 6 years may have influenced results.

The small sample size limits statistical power, particularly for detecting moderate effects. Given the final sample size of 30 participants per group, the study was adequately powered (>80% at α = 0.05) to detect large effects (d ≥ 0.8) but may have been underpowered to detect small-to-moderate effects. Consequently, non-significant findings should be interpreted cautiously, as they may reflect insufficient power rather than true null effects.

The reliance on a single geographic location and a specific patient population also limits the generalizability of our findings to broader demographic groups and diverse cultural contexts.

## Implications for practice

Despite these limitations, the findings suggest several hypothesis-generating implications for clinical practice. Long-term physical exercise programs, particularly circuit resistance training, may be considered as interventions for maintaining cardiovascular health in older adults. The observation that certain metabolic parameters showed progressively widening between-group differences suggests that sustained exercise may not only improve baseline values but may also prevent deterioration that can occur with aging and sedentary behavior. This finding, if confirmed in randomized trials, would have important implications for framing exercise as a preventive intervention.

The gradual onset of benefits observed in this study suggests that patience and persistence should be encouraged when implementing exercise programs for older adults, as improvements may take time to manifest. The incorporation of social activities both during and outside the exercise program may help increase participant engagement and long-term adherence.

The multidisciplinary approach involving various health professionals could enhance participant engagement and adherence to long-term exercise programs.

## Conclusion

This retrospective cohort analysis provides evidence that sustained participation in a 6-year circuit resistance training program may be associated with favorable cardiovascular and metabolic profiles in older adults. The gradual emergence of significant between-group differences—systolic blood pressure at 1.5 years, triglyceride-to-HDL-C ratio at 2.5 years, and triglycerides and fasting glucose at 3.0 years—highlights the potential importance of long-term exercise engagement in this population. This temporal pattern is consistent with evidence that sustained resistance training can favorably alter cardiovascular and metabolic disease markers in older adults.

The program’s longevity may be attributed to its multidisciplinary team approach and incorporation of social activities, which align with evidence-based strategies for enhancing exercise adherence in older adults. These programmatic features—including flexible attendance policies, weekly group competitions, and digital monitoring—represent potentially important elements for sustained engagement, though their individual contributions were not formally assessed.

## Data Availability

The raw data supporting the conclusions of this article will be made available by the authors, without undue reservation.
